# Perinatal lethal type II osteogenesis imperfecta: a case report

**DOI:** 10.11604/pamj.2015.21.11.6834

**Published:** 2015-05-05

**Authors:** Imene Dahmane Ayadi, Emira Ben Hamida, Rania Ben Rebeh, Sihem Chaouachi, Zahra Marrakchi

**Affiliations:** 1 Department of Neonatology, Charles Nicolle Hospital, Tunis-El Manar University, Tunis, Tunisia

**Keywords:** Osteogenesis imperfecta, newborn, prenatal diagnosis

## Abstract

We report a new case of osteogenesis imperfecta (OI) type II which is a perinatal lethal form. First trimester ultrasound didn't identified abnormalities. Second trimester ultrasound showed incurved limbs, narrow chest, with hypomineralization and multiple fractures of ribs and long bones. Parents refused pregnancy termination; they felt that the diagnosis was late. At birth, the newborn presented immediate respiratory distress. Postnatal examination and bone radiography confirmed the diagnosis of OI type IIA. Death occurred on day 25 of life related to respiratory failure.

## Introduction

Osteogenesis imperfecta (OI) is a rare inherited bone disease caused by defects in type I collagen synthesis secondary to mutations in type I collagen genes. Its prevalence is about 6-7 per 100 000 individuals [1]. Pathogenesis is related to quantitative or qualitative abnormalities of type I collagen. It consists of a heterogeneous group of skeletal dysplasia characterized by clinical variability and genetic polymorphism. Generally, there is a correlation between the clinical features and genetic mutation. OI type II is the most severe form. It is a lethal form with collagen abnormalities resulting in dwarfism, bone fragility and deformity with in utero or perinatal death [2]. There are diagnosis difficulties between OI and other skeletal dysplasia. OI type II is a severe form with early fetal skeletal defects, thus early prenatal sonographic diagnosis is possible.

## Patient and observation

**Figure 1:**
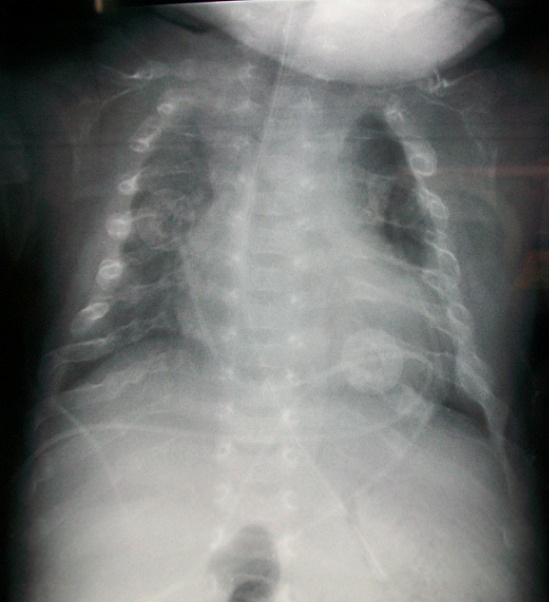
Chest: hypomineralization, narrow thorax, multiple beaded ribs

**Figure 2:**
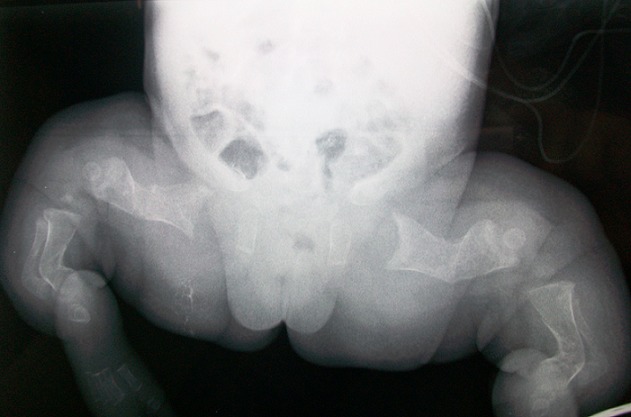
Bone demineralization; femur, tibia and fibula deformities with fractures

**Figure 3:**
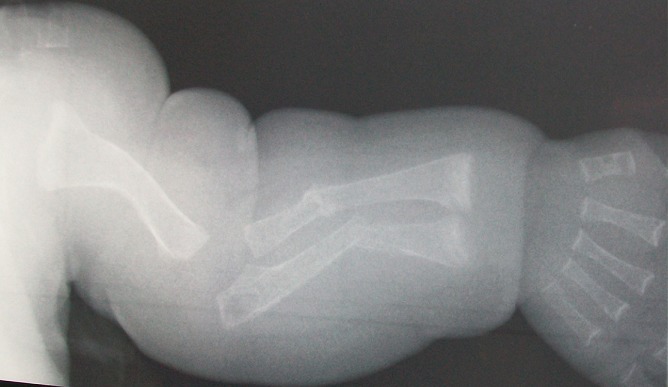
Bone demineralization; humerus deformity; radius and ulna fractures with callus formation

**Figure 4:**
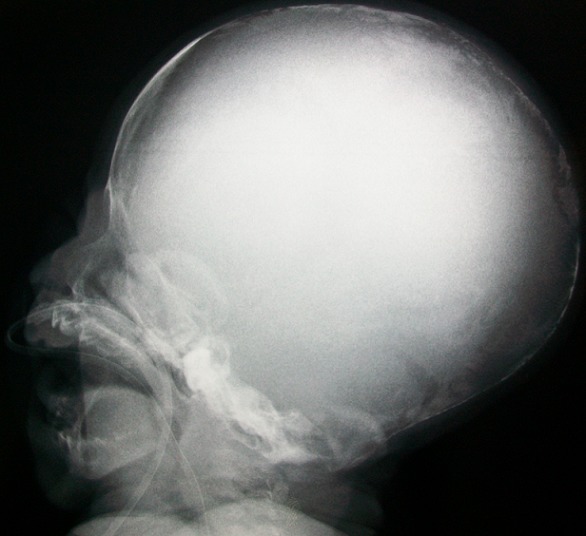
Skull: poor mineralization, wormian bones

A term female neonate born vaginally to a 29-year-old mother. There was no consanguinity, no family history of OI. Pregnancy was uneventful, prenatal laboratory test results were within reference ranges. First trimester ultrasound didn't objectified abnormalities. Prenatal ultrasound examination at 22 weeks of gestation objectified short and incurved limbs, narrow chest, with hypomineralization and multiple fractures of ribs and long bones. Interruption of pregnancy was proposed but refused by parents. Parents felt that the diagnosis was late. At birth, the newborn presented immediate neonatal respiratory distress. Postnatal examination showed macrocephaly, narrow chest, curved and shortened limbs. Postnatal bone radiography showed anomalies as follows: chest: hypomineralization, narrow thorax, multiple beaded ribs (Figure 1); limbs: bone demineralization, diaphyses deformities with fractures and callus formation (Figure 2, Figure 3); skull: poor mineralization, wormian bones (Figure 4). Based on postnatal findings, the diagnosis of OI type IIA was made confidently. Death occurred on day 25 of life related to respiratory failure, secondary to pulmonary hypoplasia.

## Discussion

Osteogenesis imperfecta (OI), also known as *"brittle-bone disease"* is an inherited disorder of bone formation [1]. The manifestations of OI consist of skeletal abnormalities such as osteopenia, fractures, deformities, and joint laxity; associated with extraskeletal features, which are highly distinctive such as blue sclerae, dentinogenesis imperfecta, hearing loss, and blood vessel fragility [3]. The prevalence of OI is about 6-7 per 100 000 individuals [1]. Severity and age at onset vary widely, from severe forms that are fatal in the perinatal period to mild forms diagnosed much later in life. A classification system that was originally proposed by Sillence and colleagues based on disease severity and progression; type II is the most severe form with perinatal death [4]. In 1984, a radiological subclassification of type II was proposed with dwarfism, lethality and multiple congenital long bone fractures present in all types. The OI type IIA is characterized by broad ribs with multiple fractures, continuous beaded ribs, severe undermodeling of femur [2]. Hydrops fetalis can be seen in association with this subtype. As it was in the present case, severe forms of OI are frequently diagnosed prenatally, as a consequence of in utero ultrasound scanning. The main classical sonographic features of OI type II include diffuse hypomineralization of the skeleton, shortening and bowing of long bones, multiple fractures [5]. It may be deformability of the skull under the passage of the probe ultrasound. In our observation, parents refused pregnancy termination; they found that the diagnostic was late. In fact, early prenatal diagnosis of OI type II is possible. Authors reported early prenatal diagnosis of OI type II by first trimester transvaginal sonography with detection of shortened curved long bones, multiple fractures and hypoechogenicity of the skeleton [6]. It is important to recognize that it can be difficult to distinguish between severe OI and other lethal skeletal dysplasias such as camptomelic dysplasia or thanatotropic dysplasia. However, ultrasound is the primary tool for prenatal screening and diagnosis of skeletal dysplasias, but in many times a precise diagnosis cannot be made. Tow-dimensional ultrasound showed limits, three-dimensional ultrasound seems to be a useful complementary method, and may improve accuracy of the prenatal diagnosis of skeletal disorders. Prenatal three-dimensional computed tomography is efficient for the diagnosis of specific fetal skeletal dysplasia. It is especially effective in confirming or excluding bone fractures, recognizing wormian bones and for detailed evaluation of the fetal spine [5, 7]. However, controversies remain about its use for prenatal diagnosis in terms of fetal radiation. Authors propose magnetic resonance imaging (MRI) for prenatal diagnosis of OI type II. MRI offer high soft tissue resolution needed to study the non-ossified fetal skeleton. In addition, it provides information about the severity of lung hypoplasia [8]. At birth, infants with OI type IIA have very short limbs curved and/or distorted (their legs are often in a frog-leg position), long bones fractures, small chest, and soft skull. The sclerae are usually blue or gray. Respiratory and swallowing problems are common. Macrocephaly may be present, microcephaly is rarely present. The radiologic features are characteristic and include absent or limited mineralization; flat vertebral bodies; very short, telescoped, broad femurs; beaded and often broad short ribs in "bamboo" [1]. The outcome of type IIA is poor with death in the perinatal period. Lethal OI is frequently associated with severe respiratory insufficiency secondary to pulmonary hypoplasia [9], as it was seen in our observation. In severe forms of OI, the occurrence of an index case, invites to offer genetic counseling to parents. This pathology is secondary to mutations in type I collagen genes. Mutations lead to a defect affecting the non-mineral part of bone responsible of severe bone fragility and defective ossification. Genetic mutations are often autosomal dominant and generally located on COL1A1 or COL1A2 (80-90%), which encode for the chains of type I collagen, the major structural protein of bone. Recessively inherited OI results in many cases from homozygous or compound heterozygous mutations in CRTAP and LEPRE1 which encode for proteins involved in collagen biosynthesis. Genetic counseling can be difficult, because more than 100 different mutations have been identified in patients with OI and novel mutations are recently found [10]. New cases of OI type II occurs frequently secondary to private autosomal dominant mutations or parental mosaicism with more genetic counseling difficulties.

## Conclusion

OI type II is a rare lethal pathology of the skeleton with severe abnormalities that may be identified by first trimester ultrasound. Lethality implies that early prenatal diagnosis is necessary to help parents making a decision relative to pregnancy termination.
